# Supplementary material for: Discovery of a Novel Simian Pegivirus in Common Marmosets (Callithrix jacchus) with Lymphocytic Enterocolitis
Source: Microorganisms. 2020 Sep 30;8(10):1509. doi: 10.3390/microorganisms8101509 (PMC7599636; doi:10.3390/microorganisms8101509)
Supplement: Supplementary file 1 [file microorganisms-08-01509-s001.pdf]

microorganisms

MDPI

Table S1. Association of SOBV with pathology in 81 common marmosets at the WNPRC.

Organ or organ system	Total number of marmosets with pathology	Number of marmosets infected	Number of marmosets non-infected	*p* value (univariate logistic regression)
HEENT	5	3	2	0.317
Central nervous system	3	1	2	0.858
Peripheral nervous system	1	1	0	0.996
Heart	14	6	8	0.698
Lungs	22	6	16	0.218
Blood vessels	4	1	3	0.581
Esophagus	2	0	2	0.996
Stomach	5	2	3	0.935
Intestines	66	28	38	0.118
Kidneys	64	26	38	0.401
Reproductive system	18	9	9	0.249
Urinary system	2	1	1	0.732
Liver	52	22	30	0.319
Gallbladder	9	3	6	0.747
Skin and hair	8	4	4	0.476
Lymphatic system	56	21	35	0.831
Spleen	21	10	11	0.308
Bone marrow	11	3	8	0.424
Muscle	5	4	1	0.0830
Bone	2	2	0	0.996
Thyroid	18	7	11	0.951
Parathyroid	8	5	3	0.152
Pituitary	2	1	1	0.732
Adrenal	49	20	29	0.560
Thymus	4	2	2	0.624

© 2020 by the authors. Submitted for possible open access publication under the terms and conditions of the Creative Commons Attribution (CC BY) license (http://creativecommons.org/licenses/by/4.0/).
